# Supplementary figures and images for: Interpretable machine learning with tree-based shapley additive explanations: Application to metabolomics datasets for binary classification
Source: PLoS One. 2023 May 4;18(5):e0284315. doi: 10.1371/journal.pone.0284315 (PMC10159207; doi:10.1371/journal.pone.0284315)

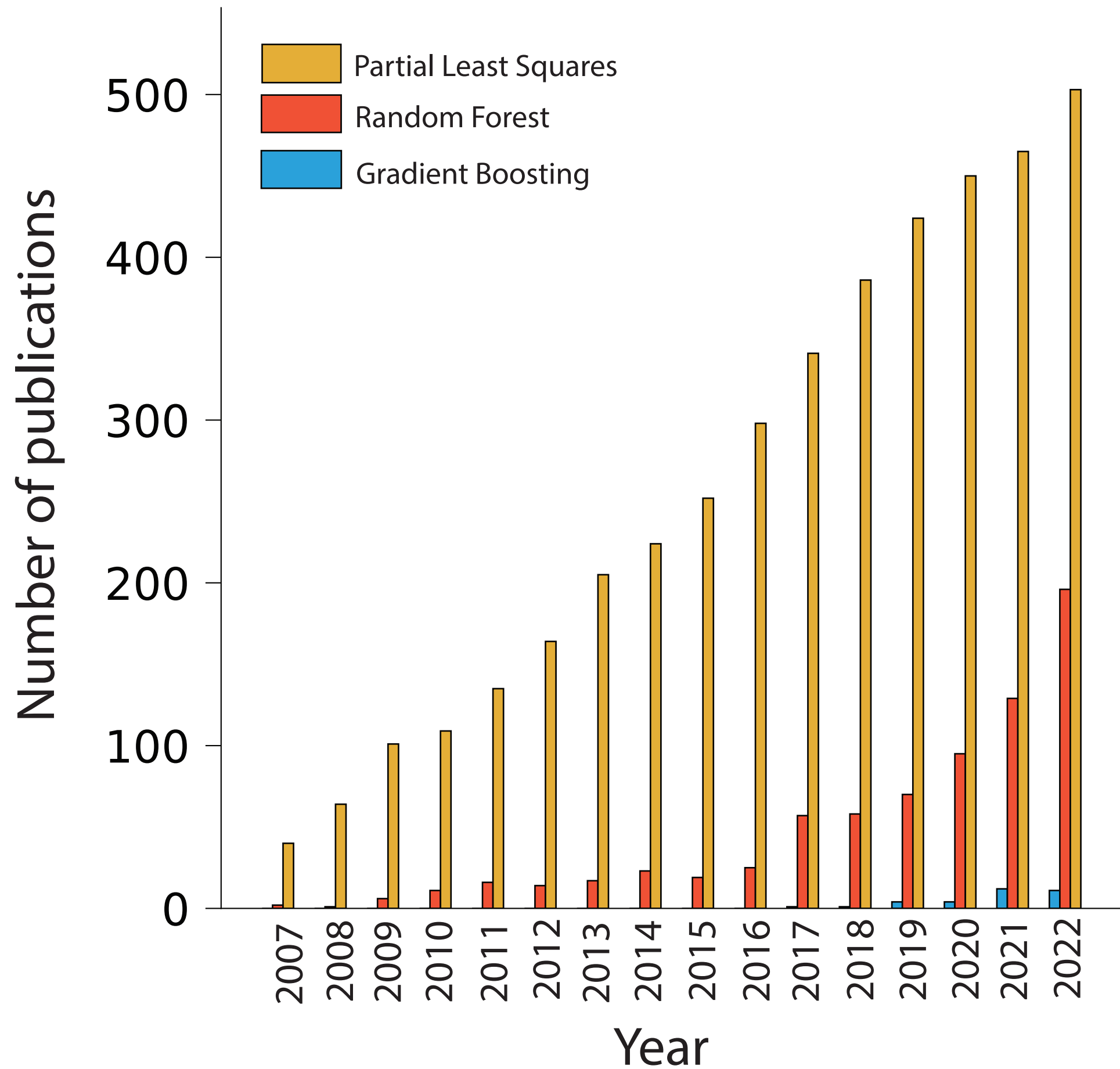

Supplement: S1 Fig — The data shown is from 2007–2022. For partial least squares regression, the following keywords were used for the search as described in the method section: ("Metabolomics"[Mesh] OR metabolite* OR metabolom* OR metabonom*) AND ("Partial least squares*" OR "PLS"). For random forest: ("Metabolomics"[Mesh] OR metabolite* OR metabolom* OR metabonom*) AND ("random forest*"). For XGBoost and gradient boosting: ("Metabolomics"[Mesh] OR metabolite* OR metabolom* OR metabonom*) AND ("extreme gradient boosting" OR "gradient boosting"). (PDF) [file pone.0284315.s001.pdf]

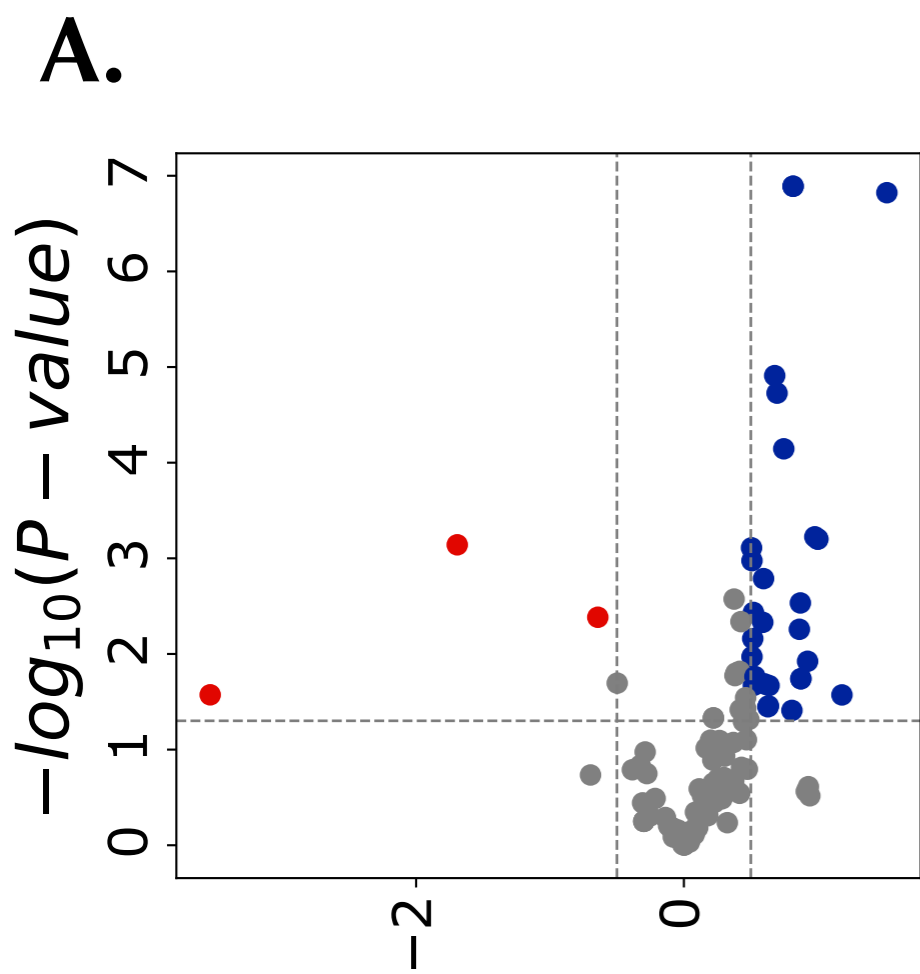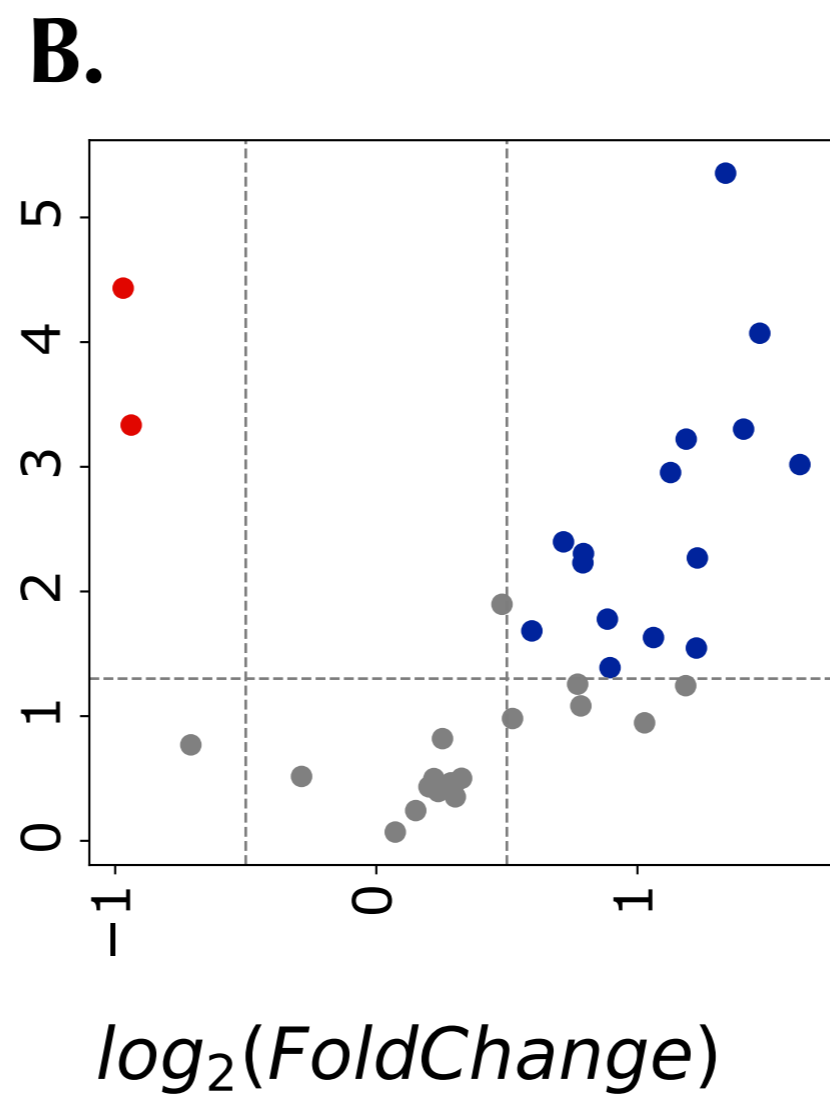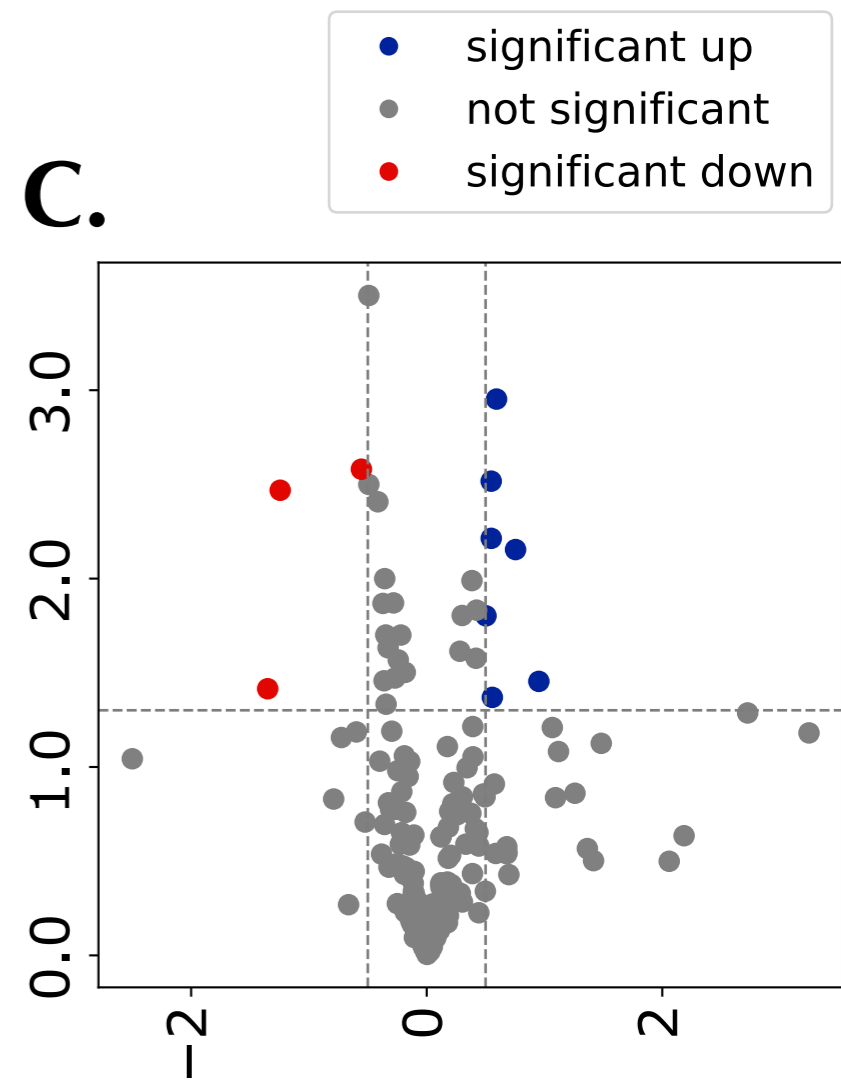

Supplement: S2 Fig — (A), MTBLS404, (B) MTBLS547, (C) ST000369. Significant up (blue) are the metabolomic features that are higher in the males for MTBLS404, high-fat diet fed mice for MTBLS547, and adenocarcinoma lung cancer patients in ST000369. Significant down (red) are the metabolomic features that are higher in the females for MTBLS404, normal diet fed mice for MTBLS547, and healthy controls in ST000369. (PDF) [file pone.0284315.s002.pdf]

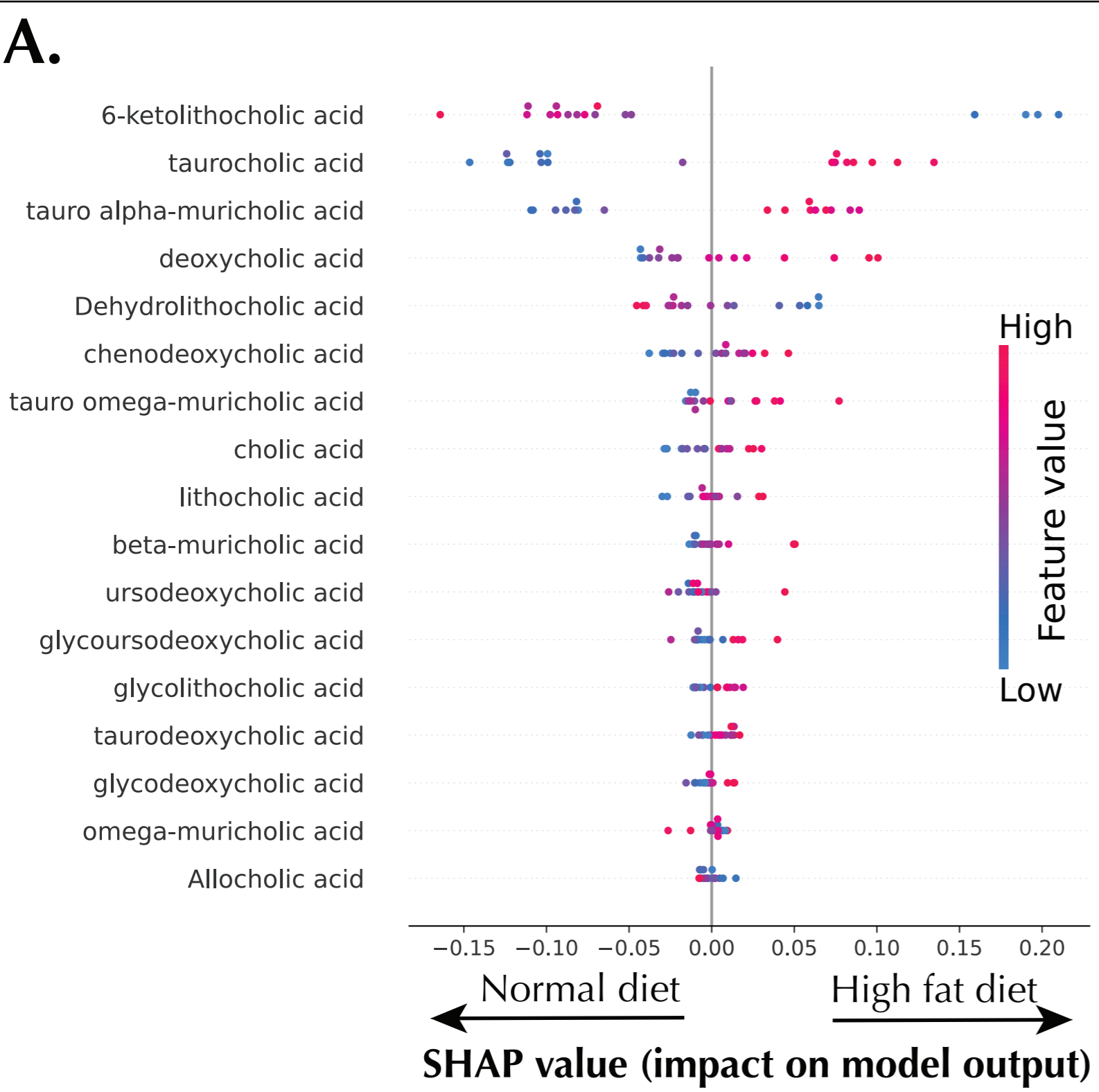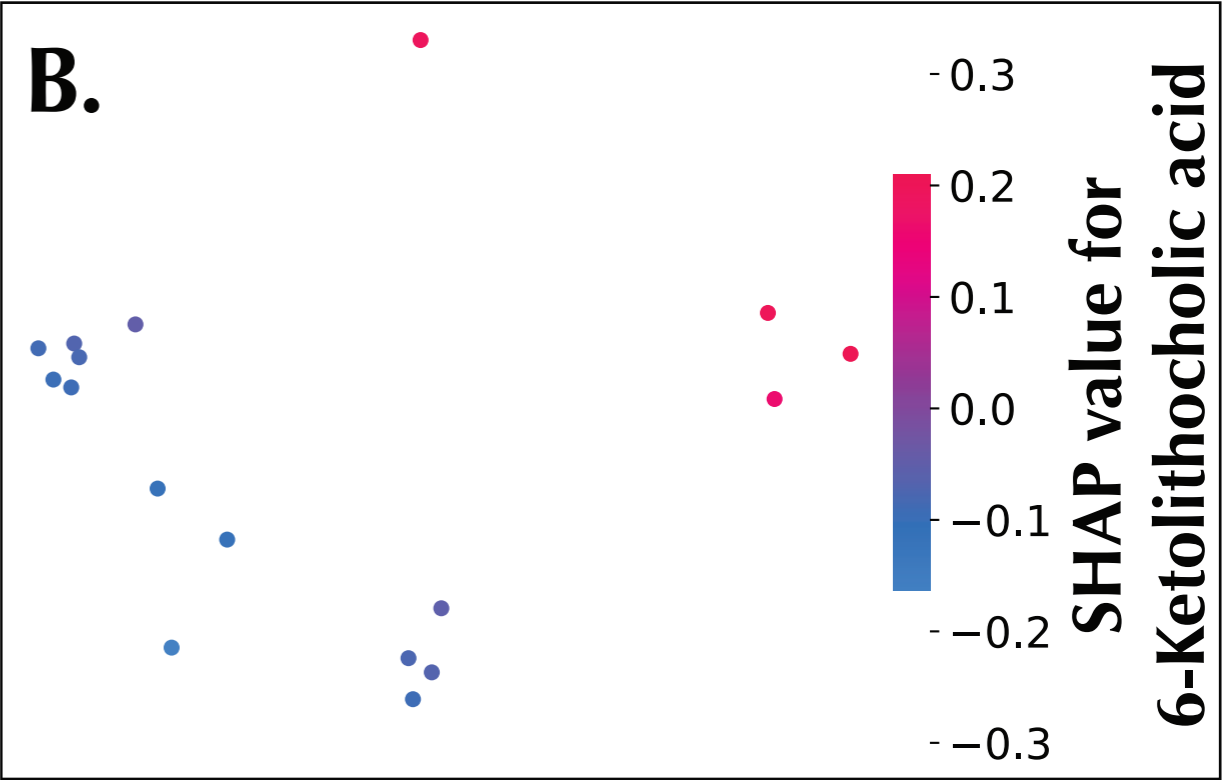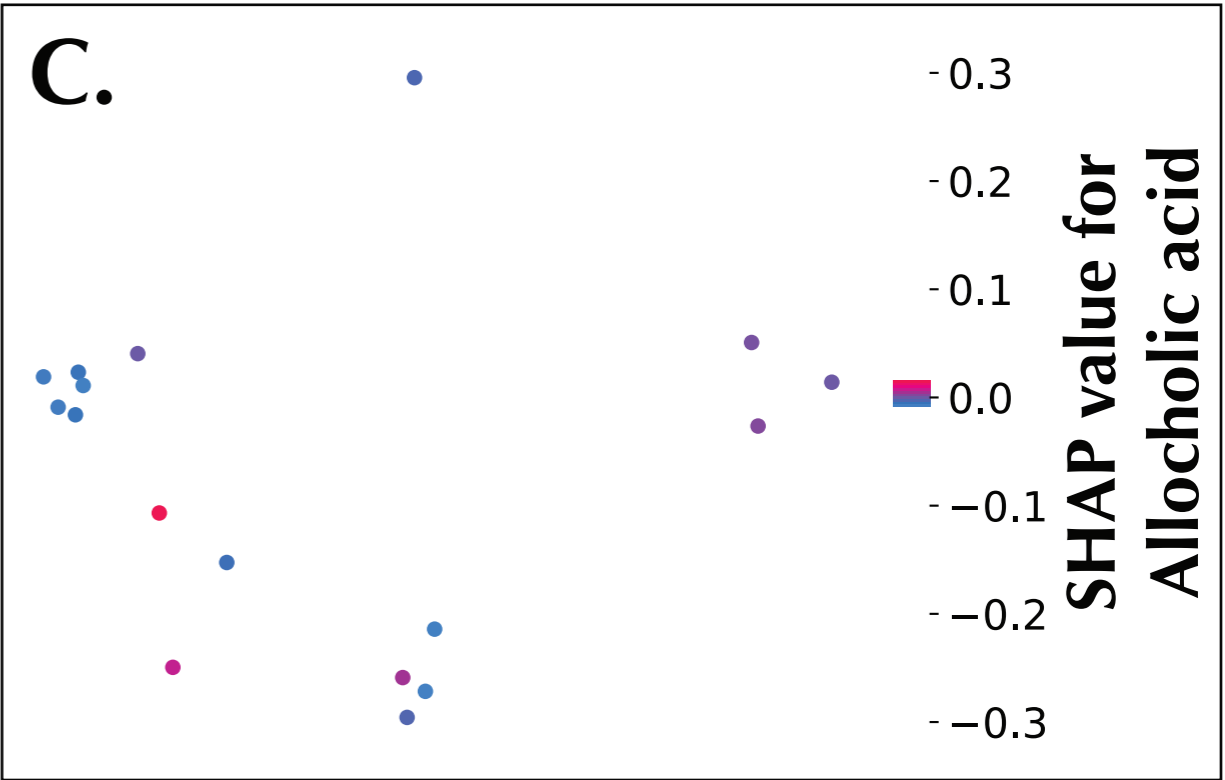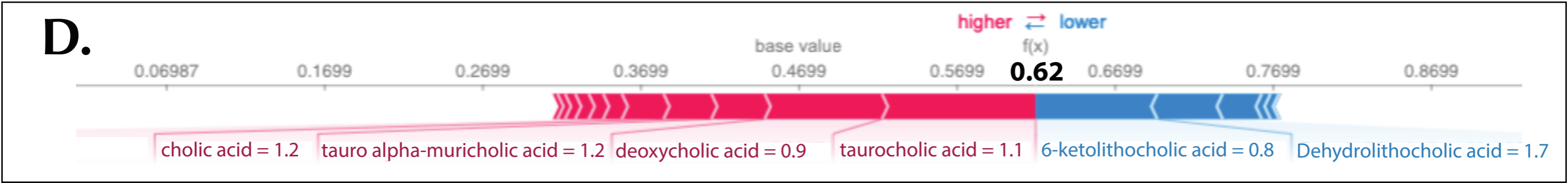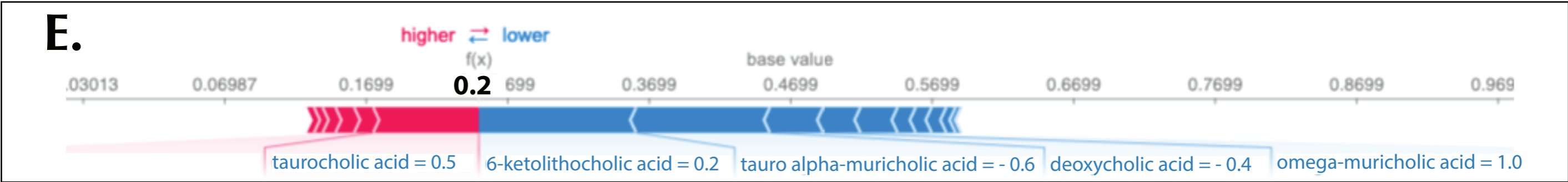

Supplement: S3 Fig — A: SHAP summary plot for MTBLS547 test set. B: SHAP embedding plot for 6-ketolithocholic acid. C: SHAP embedding plot for Allocholic acid. D: Force plot showing the interpretation of a true positive sample. E: Force plot showing the interpretation of a true negative sample. In this interpretable machine learning analysis, the impact of a high-fat diet (HFD) on bile acids in the mice cecum is presented. True positive indicates the correct prediction of mice on HFD. True negative indicates the correct prediction of mice on a normal diet. 6-ketolithocholic acid (6-ketoLCA) is the most important metabolomics feature in the model, with low feature values of 6-ketoLCA associated with HFD and high feature values associated with a normal diet. The least important feature is Allocholic acid. SHAP values were projected in a 2-dimensional space via PCA, which can be used to visualize the impact of a metabolomic feature as observed in both the intensity of the SHAP values and the clustering of positive and negative SHAP values, respectively. (PDF) [file pone.0284315.s003.pdf]

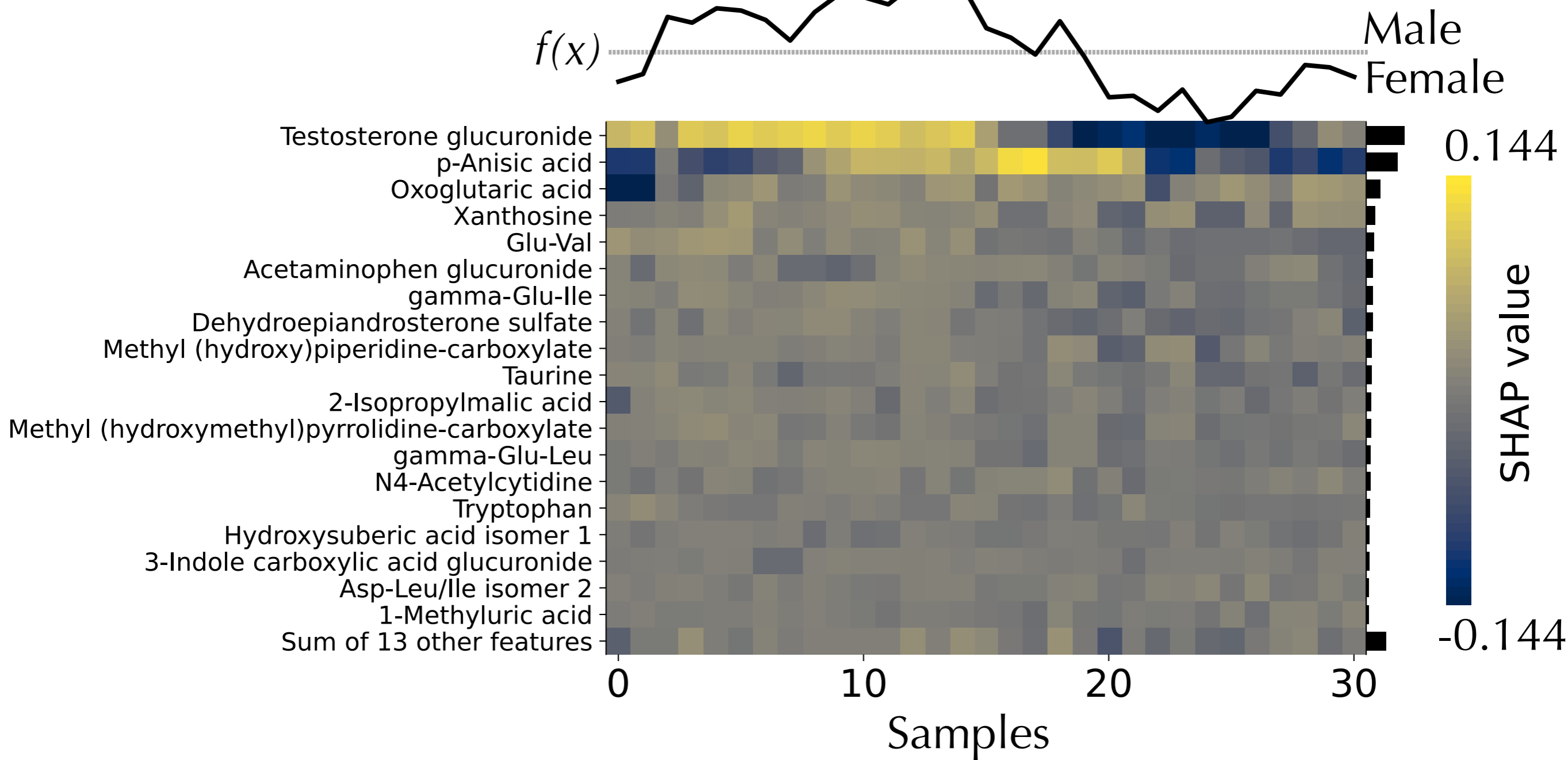

Supplement: S4 Fig — Samples are displayed on the x-axis, while features are arranged in ascending order of importance on the y-axis. f(x) indicates the prediction outcome, with the line plot over the dotted line indicating male predictions, while the line plot below the dotted line indicates female prediction. The bar plot represents the mean absolute SHAP value, the average impact on the model output. (PDF) [file pone.0284315.s004.pdf]

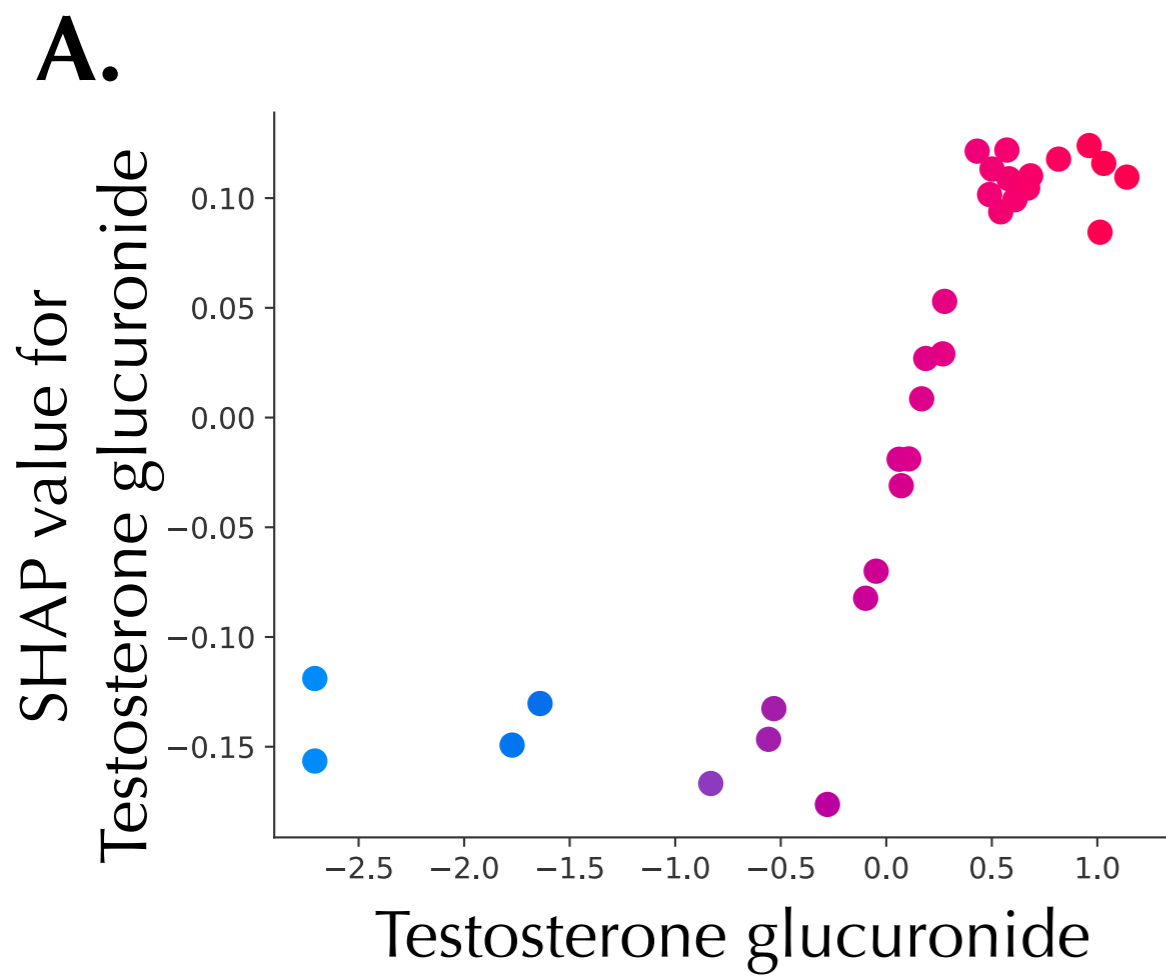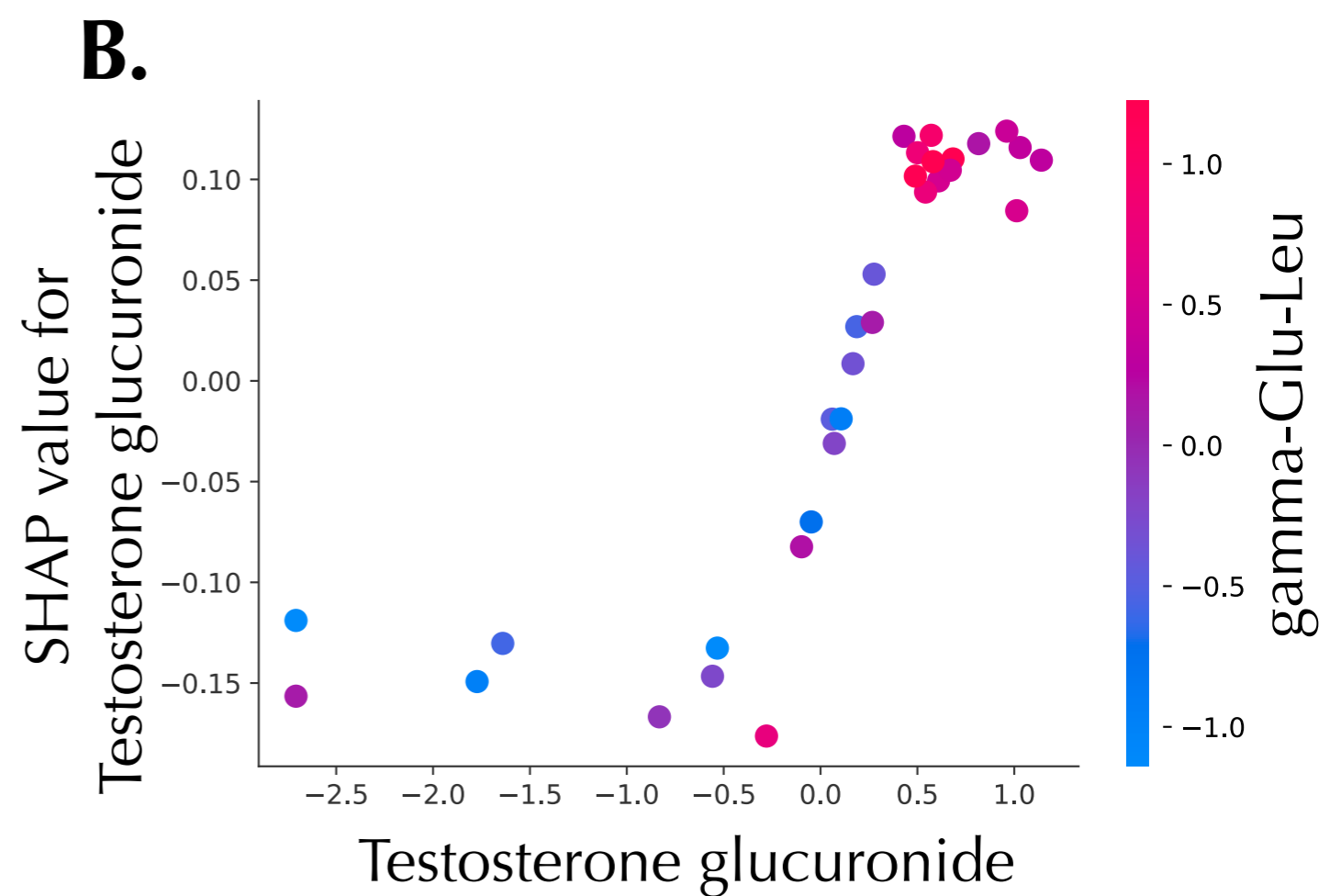

Supplement: S5 Fig — A) Testosterone glucuronide. B) Testosterone glucuronide and γ-glu-leu. (PDF) [file pone.0284315.s005.pdf]
